# Aberrant expression of B7-H3 in gastric adenocarcinoma promotes cancer cell metastasis

**DOI:** 10.3892/or.2022.8421

**Published:** 2022-10-03

**Authors:** Wei Dai, Genhai Shen, Jianping Qiu, Xin Zhao, Quangen Gao

Oncol Rep 32: 2086–2092, 2014; DOI: 10.3892/or.2014.3405

Following the publication of the above paper, an interested reader drew to the authors' attention that the scratch-wound assay data featured in Fig. 5A on p. 2090 appeared to contain some overlapping data comparing between the panels, such that these data, which were intended to show the results from differently performed experiments, may have been derived from the same original source. After having re-examined their original data, the authors conceded that the figure had been assembled incorrectly (specifically, four of the images selected out of the nine data panels included in this figure part had been chosen erroneously). Furthermore, the first author on the paper (Wei Dai) also acknowledged that several of the named authors had not given their prior consent to be included as such as authors on the paper, and therefore a request was made that the paper be retracted from the publication.

In view of these admissions, the Editor of *Oncology Reports* has accepted the authors' request that the paper be retracted from the journal. The Editor apologizes to the readership for any inconvenience caused.

